# Super-resolution measurement of distance between transcription sites using RNA FISH with intronic probes

**DOI:** 10.1016/j.ymeth.2015.11.009

**Published:** 2016-04-01

**Authors:** Joshua D. Larkin, Peter R. Cook

**Affiliations:** Sir William Dunn School of Pathology, University of Oxford, South Parks Road, Oxford OX1 3RE, UK

**Keywords:** Nascent intronic RNA FISH, Super-resolution localization, Transcription location, Diffraction limit, Colocalization, Long gene, Inner-nuclear distance

## Abstract

Nascent transcripts being copied from specific human genes can be detected using RNA FISH (fluorescence *in situ* hybridization) with intronic probes, and the distance between two different nascent transcripts is often measured when studying structure–function relationships. Such distance measurements are limited by the resolution of the light microscope. Here we describe methods for measuring these distances in cultured cells with a precision of a few tens of nanometers, using equipment found in most laboratories (i.e., a wide-field fluorescence microscope equipped with a charged-coupled-device camera). Using images of pairs of transcripts that are often co-transcribed, we discuss how selection of cell type, design of FISH probes, image acquisition, and image processing affect the precision that can be achieved.

## Highlights

• Label intronic RNA using FISH to identify sites of transcription by RNA polymerase II.
• Low-tech microscopes are used to acquire images for super-resolution measurements.
• Distances between sites of transcription are determined with precision near 20 nm.
• Spatial-temporal relationships between active genes are studied with this method.

## 1 Introduction

When and where human genes become active in the nucleus, and the relationship between activity and chromosome conformation, are currently areas of great interest [1]. Chromosome conformation capture (3C) [2] and fluorescence *in situ* hybridization (FISH) [3] are the two methods that are in the widest use for localizing one gene relative to another. Both 3C and FISH applied with a probe targeting a gene (i.e., DNA FISH) are unable to distinguish if the gene in question is active or not, so other experimental approaches must be used to determine activity. However, FISH applied with a probe targeting intronic RNA (i.e., RNA FISH) [4] can be used to localize the nascent transcript (and so an active gene) if it is assumed that introns are found only at sites of transcription [5]. This assumption is broadly true, as most introns are removed co-transcriptionally [6] and then degraded quickly with half-lives of ∼5 min [7]. Consequently, RNA FISH is often the technique of choice for localizing nascent transcripts (and so the genes that encode them).

Localizing a FISH signal within the nucleus presents several major challenges. First, any technique that uses a light microscope is limited by the wavelength of the light used during imaging [8]; consequently, the location of a molecule is usually determined to within hundreds of nanometers. However, investigators are often interested in the molecular interactions that their gene of interest might make, and so would like to localize signals to within a few nanometers. Second, the nucleus contains few landmarks (the main ones being the periphery, nucleoli, and clumps of heterochromatin), and investigators are usually interested in localizing their signal relative to other features like a specific chromatin segment (perhaps tagged with a fluorescent protein or antibody), or another FISH signal (which might mark a different gene or transcript). Consequently, absolute measurements of position are usually of less interest than relative ones.

Here, we discuss methods used to determine relative distances between nascent transcripts, down to distances of several tens of nanometers. We will not discuss the use of sophisticated “super-resolution” microscopes, as this is amply discussed in the rest of this volume; instead, all experiments described involve a standard fluorescence microscope of the kind found in most cell-biology laboratories. To provide focus, we will often use as an example the activation of one particular human gene (i.e., *SAMD4A*) in one particular cell type (i.e., human umbilical vein endothelial cells, HUVECs) by one particular cytokine (i.e., tumour necrosis factor α, TNFα). This system has various advantages in this context [9]. First, *SAMD4A* is 221 kbp long, and this great length allows the technique used to assess proximity in nuclear space to be applied with high precision. Second, HUVECs are diploid and – in the cases discussed – synchronized in G0 phase, so there are no complicating effects of additional gene copies. As these cells are also being studied in detail by the ENCODE project [10], we know which transcription factors are bound in and around *SAMD4A*, and which histone marks are associated with the gene before activation. They can also be obtained from pooled or single donors (Lonza), so allowing the study of cells with homogenized or unique genetic backgrounds. Third, we have detailed knowledge of the first transcription cycle following activation by TNFα, a well-studied cytokine that orchestrates the inflammatory response [11]. In most experiments discussed, *SAMD4A* is initially inactive, as the relevant transcription factor – nuclear factor κB (NFκB) – is sequestered in the cytoplasm. However, when TNFα is added, NFκB floods into nuclei and facilitates initiation by a “pioneering” polymerase within ∼10 min. This pioneer then continues to transcribe this long gene (at ∼3 kbp/min) until it reaches the terminus after another ∼75 min. As initiation is reasonably synchronous in the cell population, and as polymerases on different *SAMD4A* genes transcribe at much the same rates, sampling after 0, 10, 30, 60 and 85 min allows one whole transcription cycle to be monitored in the population. Detailed information on the binding of RNA polymerase II comes from ChIP and ChIP-seq [12], [13], on the changing levels and half-lives of nascent RNAs from tiling microarrays, RNA-seq, RNA FISH, and RT-PCR [12], [14], [15], [16], [18], on histone modifications from ChIP-seq [12], on nucleosomal rearrangements from MNase-seq [19], and on the binding of NFκB from ChIP-seq [14], [17]. In summary, this system provides an excellent molecular switch; on stimulation with TNFα, the number of cells with at least one active *SAMD4A* allele (assessed by RNA FISH) increases from <3% to ∼70% over 30 min [9], [13], [14], [15].

We now describe the various factors that influence the resolution that can be obtained when colocalizing transcripts using RNA FISH and a standard fluorescence microscope.

## 2 Overview of the method

**Fig. 1:**
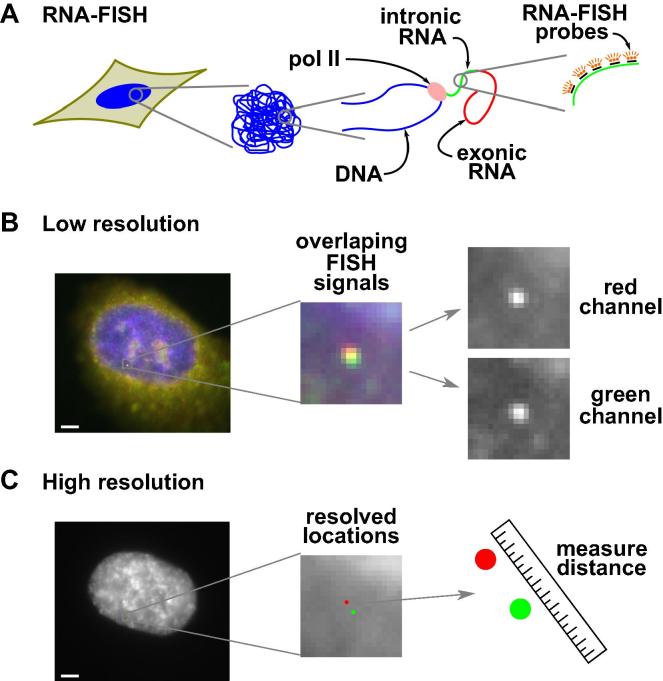
Diagrammatic summary of the method. (A) Identify sites of transcription using RNA-FISH by targeting intronic RNA. (B) Resulting images are of nuclei that contain diffraction-limited spots (∼200 nm). Desired measurement resolution is on the order of tens of nanometers. Increased resolution is attained by localizing signals with high precision (∼20 nm) through the mapping of the precise location of the point source of light within the image of a diffraction-limited spot. Spots in each channel are isolated from the larger image for localization. (C) Distances between signals can then be assessed with high resolution. Bar: 2 μm.

This method involves labeling intronic regions of nascent RNA (Fig. 1A), to enable spatial information about gene transcription to be deduced [4]. In a typical experiment, cells are grown on coverslips before stimulation with TNFα, which switches on *SAMD4A*. The amount of time required for the target region to be transcribed is allowed to elapse before cells are fixed. Care is taken to preserve unstable intronic RNA while FISH probes are allowed to hybridize to their targets and cells are mounted for imaging. Imaging is conducted using a standard wide-field fluorescence microscope. Super-resolution measurements are made by, first, identifying RNA-FISH signals (Fig. 1B). Next, selected signals are analyzed so as to mathematically estimate the location of the signal with high precision. Here, we discuss the use of the most popular method of analysis, and discuss the advantages of other methods. Finally, following localization of transcription sites, relative distances can be measured and subsequent data analysis performed (Fig. 1C).

## 3 General considerations

When localizing mRNAs, probes usually target exons. Here, however, we are concerned with localizing nascent RNAs being transcribed from single-copy genes, and – as <1% mRNA is found at a transcription site [5] – this means that it is not feasible to use exonic probes (because the other 99% provide too high a background). Therefore, probes should target introns [4]. Obviously, they should also be bright enough to be imaged (generally at least 3× brighter than the background), and the imaging equipment (i.e., light sources, chromatic filters, and cameras) should be selected to maximize signals relative to the usual culprits that contribute to noise (e.g., auto-fluorescence, channel bleed-through when different colors are being imaged, camera noise). Perhaps surprisingly, this does not necessarily mean that the latest and most expensive microscope in your facility should be used – which is usually in great demand. For example, we used a 10-y old microscope/camera system to obtain all the results discussed here, and found that our time was best spent on optimizing conditions on a little-used microscope.

RNA-FISH probes should target regions short enough to form a spatial arrangement smaller than the diffraction limit of light (∼200 nm), so as to behave similarly to a point-source of light. As a transcript of ∼600 nucleotides has a contour length of ∼200 nm, and as transcripts can be expected to be folded in the cell, all complementary sequences targeted by the probe set should therefore lie within ∼600 nucleotides (ideally <400 nucleotides). For super-resolution measurements, the intent is to acquire images of spots that resemble an Airey disk – the shape produced by a point source of light when viewed in a microscope. Patterns larger than a point source increase measurement uncertainty.

Imaging can be conducted using 2D or 3D imaging modalities. For our purposes, it has been sufficiently accurate to image and conduct 2D measurements, accounting for unknown distances in the third dimension as error in our distance measurements. However, other studies may require 3D imaging. Whichever imaging modality is used, it is important that attention be paid to inter-channel alignment. Chromatic aberrations are corrected for in many microscope objectives, but residual misalignment can still produce error greater than the distances to be measured.

## 4 Detailed protocol

This protocol is based on the standard ones developed by others [3], [4], [20], [21], [22], adapted for our specific purposes as indicated.

### 4.1 Design of RNA-FISH probes

RNA FISH probes comprise two components: the DNA bases complementary to the RNA target and the attached fluors. Probe design involves balancing many factors: target specificity (all probes interact non-specifically to some extent with non-target molecules), imaging brightness, and compact geometry (to allow easy access to targets). Twenty-mers are long enough to hybridize uniquely with most targets in the human transcriptome, and 20–50-mers (each labeled with a single flour) are often used [20]; longer oligomers increase specificity and hybridization strength [21]. This is advantageous because it allows more stringent washing (which decreases non-specific labeling). We describe the use of ∼50-mers (which are still small enough to penetrate fixed cells efficiently), but many of the same arguments apply to ∼20-mers.

Oligonucleotides with the usual thymine residue replaced by an amino-modified one can be made reasonably cheaply in oligonucleotide synthesizers. Consequently, it is often convenient to buy oligonucleotides bearing such modified bases (i.e., with amino-modifier C6-dT nucleotides) and then add a fluor of choice (e.g., Alexa 555 or Alexa 647) using a commercially-available kit (i.e., one containing a fluor with an attached chemical group that allows conjugation of the fluor to the amino group) [21], [22]. Alternatively, 50-mers with pre-attached fluors can be purchased directly. As two fluors attached to bases in an oligonucleotide only a few bases apart can quench fluorescence (exciting energy is transferred between the two without emission of a photon), modified bases should be spaced more than ∼8 bases apart [21], [22]. Consequently, ∼5 modified bases are usually included in a 50-mer. Unfortunately, chemical coupling may then result in only 3 or 4 fluors becoming attached to the 50-mer. To increase signal, sets of 5 50-mers (giving a total of ∼25 fluors) are often used [21], [22]. To ensure that each oligomer in a set hybridizes as efficiently as the others, all are chosen so they have roughly the same GC content (usually ∼55%).

In the examples described, we use sets of 5x50-mers bearing amino-modifier C6-dT nucleotides (each with a sequence know to hybridize well in ‘tiling microarrays’ bearing 25-mers) [12]. [A ‘good’ oligomer is one that gives a strong microarray signal with RNA from cells treated with TNFα (i.e., when the gene is being transcribed) compared with RNA from untreated cells (i.e., when the gene is ‘off’). This allowed us to eliminate oligomers that happened to hybridize non-specifically with transcripts from other parts of the genome. As such microarray data is usually not available, it is sufficient to choose 50-mers that lack any 20-nucleotide segments that map to other regions of the genome.] These oligonucleotides were synthesized commercially (e.g., Gene Design, Japan), and fluors (e.g., Alexa Fluor 488, 555, or 647; Invitrogen) coupled on to them using a kit (Invitrogen). This choice of fluors leaves the green channel available for detection of the green fluorescent protein if needed, and – as background due to autofluorescence is higher at shorter wavelengths – this choice ensures a usable signal-to-noise ratio. Probes were then purified to remove unattached fluorescent molecules using G-50 columns (GE Healthcare), ethanol precipitated twice, concentrated using a Microcon-30 column (Millipore), and labeling efficiencies calculated using the Base:Dye ratio calculator (Invitrogen) [23]. Probes were stored in aliquots at −25 °C in a dark container until use, so as to limit degradation from freeze-thaw cycles and photo-bleaching from exposure to ambient light.

### 4.2 Coverslip preparation

Coverslips can be prepared in any manner that removes debris and contaminants while rendering the surface suitable for cell adhesion and growth (debris complicates image acquisition and can add to noise via auto-fluorescence, while contaminants may hinder cell growth). The ability of a cell to adhere to a surface depends on the cell type used. We commonly use #1.5 (22 × 22 mm) coverslips and sonicate them in 1% hydrofluoric acid for 5 min. Sonication agitates the surface so as to release debris and contaminants; hydrofluoric acid dissolves many materials, including glass, and so leaves the surface clean and slightly etched. [Exceptional safety precautions must be taken when using hydrofluoric acid.]

Following sonication, coverslips are rinsed 10–20 times in sterile (e.g., ‘milli-Q’) water. For this step, it is efficient to transfer coverslips between beakers of sterile water using a coverslip rack made of Teflon (e.g., Coverslip Mini-Rack, C-14784; Molecular Probes). Coverslips are stored in absolute ethanol in a sealed container so as to prevent evaporation. Immediately prior to use, coverslips are quickly flame dried to sterilize them and remove all residual ethanol.

### 4.3 Cell culture

The cells that are the most suitable for high-resolution imaging are generally the ‘flattest’ – ones giving the closest to a 2D profile. In the cases described here, HUVECs were obtained from pooled donors (Lonza), grown to 80–90% confluence in Endothelial Basal Medium 2-MV with supplements (EBM; Lonza) and 5% FBS. Prior to imaging, cells were plated on clean coverslips and allowed to grow to ∼90% confluence, ‘starved’ for 16–18 h in EBM+0.5% FBS (this halts cells in the G1 phase of the cell cycle, so essentially all cells in the population contain only two copies of *SAMD4A*), treated ± TNFα (10 ng/ml; Peprotech), and grown for different times.

### 4.4 Fixation

The purpose of fixation is to preserve as much structure as possible, so the fixative used should be the one that achieves this best with the cells being analysed. For RNA FISH, preserving RNA integrity is an additional major issue; RNA is prone to hydrolysis both spontaneously and enzymatically. Therefore, the usual precautions are applied to ensure all solutions, containers, and pipet tips are free of RNase. Gloves should be used and changed frequently, glassware and metalware should be baked (230 °C for 2 h) or treated with RNaseZap reagent (Invitrogen), plastic consumables should be from previously unopened containers or certified RNase-free, and water and buffers should be treated with diethylpyrocarbonate (DEPC), except for reagents containing amine groups (e.g., Tris, HEPES) which should be treated with RNAsecure reagent (Invitrogen).

In our case, HUVECs grown on coverslips were fixed (17 min; room temperature) in 4% paraformaldehyde/0.05% acetic acid/0.15 M NaCl, washed 3× in PBS, permeabilized (5 min; 37 °C) in 0.01% pepsin in 10 mM HCl (pH 2.0), rinsed in water treated with DEPC, post-fixed (5 min; 20 °C) in 4% paraformaldehyde/PBS, and stored (overnight; −20 °C) in 70% ethanol. Just prior to adding FISH probes, fixed cells on coverslips should be dehydrated serially in 70%, 80%, 90% and absolute ethanol.

### 4.5 Probe hybridization and sample mounting

A small amount of labeled probes (e.g., 25 ng) are mixed with ‘hybridization mixture’ – a concoction intended to facilitate hybridization while blocking non-specific interactions between probes and cells; it contains 25% deionized formamide, 2× SSC (Sigma–Aldrich), 250 ng/ml sheared salmon sperm DNA, 5× Denhardt’s solution (Thermo Fisher), 50 mM phosphate buffer, and 1 mM EDTA (pH 7.0). The mixture plus probe is now added to coverslips, overlaid with another coverslip, to prevent dehydration and limit volume reduction, and incubated to allow hybridization between probes and targets overnight (37 °C in a moist chamber). Next, cells on coverslips were washed once in 4× SSC (15 min) and three times in 2× SSC (10 min), at a temperature intended to remove probes bound non-specifically (37 °C). Finally, coverslips were mounted in Vectashield (Vector Laboratories) supplemented with 1 μg/ml DAPI (4,6-diamidino-2-phenylindole; Sigma) to stain DNA, and slides stored at 4 °C until imaged.

### 4.6 Image acquisition

**Fig. 2:**
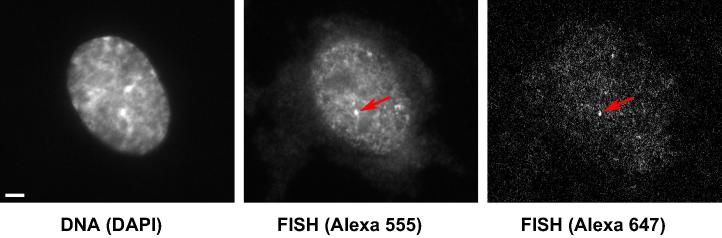
Unprocessed Images. DNA is stained with DAPI for reference. Typical FISH spots are diffraction-limited in size (∼200 nm), and must be of adequate signal-to-noise ratio to be localized with high precision. RNA-FISH signals of typical 50-mer probes, as described in the text, can be bright (e.g., Alexa 555 shown) but are often not (e.g., Alexa 647 shown). Identification of FISH spots is often difficult at the time of acquisition and requires long exposure times and post-acquisition processing. Noise sources include auto-fluorescence and non-specific probe binding. Bar: 2 μm.

Typically images were collected within 24 h of mounting coverslips, so as to minimize sample degradation and signal loss. We chose to acquire images using a common widefield fluorescence microscope – an Axioplan 2 inverted microscope (Zeiss) fitted with a CoolSNAP*_HQ_* camera (Photometrics) running under MetaMorph 7.1 software (Molecular Devices). With newer camera technology (e.g., back-thinned EMCCD, SCMOS) one can expect reduced image noise, and so increased localization precision (see below). Imaging filters should be carefully selected (we found it helpful to use a software-based chromatic selection tool [24] to minimize bleed-through of light from probes into unwanted channels). The flours used in the example study described herein were Alexa647 and Alexa594. To image Alexa647, the following excitation, dichroic splitting, and emission filters were used: 650-13, 660, 684-24 (Semrock). For Alexa594: 580-23, 593, 615-20. While Alexa 594 produced sufficiently bright signal, Alexa 647 was always faint (Fig. 2). Signal brightness could likely be improved by using a camera more sensitive at this wavelength. Adequate channel separation is verified by imaging fields of spectrally distinct fluorescent polystyrene beads (Invitrogen).

The microscope was fitted with a 63×/1.45 NA objective; use of a high numerical aperture minimizes spot size, thereby increasing localization accuracy (more on this below). The objective was optically corrected for chromatic and spherical aberration, but residual aberrations exist and give inter-channel misalignment. Because this misalignment is largely caused by microscope optics, it remains consistent between images, and so a single image of beads can be used to correct all other images acquired under the same conditions (details below). Typically, to be safe, we would acquire an image of beads each day we acquired a new image set.

Initially, we found it difficult to identify fluorescent foci given by FISH probes, even with ∼25 fluors in a probe set, simply because signals are so faint (examples of unprocessed images are illustrated in Fig. 2). We would often acquire many images in a blind yet systematic manner to ensure the same region of coverslip was not imaged more than once, before identifying foci post acquisition. The biggest challenge this presents is ensuring the imaging plane remains in focus. This can be achieved using the DAPI channel for initial, coarse, focusing, and then signal from autofluorescence and non-specifically bound labels in one of the probe channels for fine focusing.

Images are typically captured with a long exposure (10–60 s) to maximize signal brightness (and so localization accuracy). However, this causes fluor bleaching, so care should be taken not to inadvertently image the same region of the coverslip more than once.

### 4.7 Image processing and super-resolution measurement

Foci (i.e., regions of images that contain signal from a single probe set) were initially selected manually, then checked by a computer algorithm to meet criteria for a diffraction-limited spot (i.e., they should possess a Gaussian-like shape, adequate signal-to-noise ratio, and adequate local contrast). There exist a few methods for the estimation of the position of a point light-source giving a diffraction-limited spot; the most common involves statistically fitting a 2D Gaussian intensity profile to the image of a focus using regression analysis to minimize least squares error [25]. Other methods include use of the JD algorithm [13], centroid estimation [26], and maximum-likelihood estimation [27]. Each method yields an estimate of the location of the source of light (i.e., the fluors bound to the RNA) with a precision that is an order of magnitude more precise than the diffraction limit of light of ∼200 nm. Each method offers its own advantages in terms of accuracy, robustness amongst noise, and ease of use (for more detail on the topic, see Larkin and Cook, 2012 [13]). We have used all these methods (implemented using custom-written programs in MATLAB; MathWorks; available for download at [28]), and found that maximum-likelihood estimation is the most accurate, but at the cost of ease-of-use; it also requires significant computation time. Centroid estimation is the easiest to implement and is reasonably accurate when signal-to-noise ratio is high (i.e., when foci are ‘bright’), but rapidly breaks down in noisy images. The JD algorithm is more accurate than centroid estimation when signal-to-noise ratio is low, and nearly as easy to implement; it is also nearly as accurate as maximum-likelihood estimation, whilst being much faster to compute. Nevertheless, minimizing least squares remains the most popular method, probably because it was the first to be used for this purpose and it remains mathematically intuitive – it is the same method we think of using when fitting a line to a scatter plot.

**Fig. 3:**
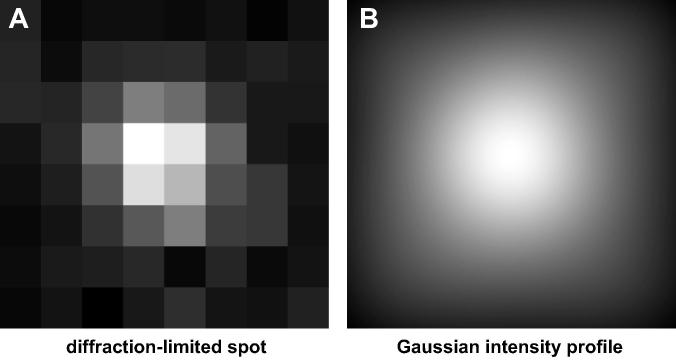
Location estimation via fitting a Gaussian-shaped intensity profile to an image of a diffraction-limited spot. (A) An image of a diffraction-limited spot as acquired from a camera provides inadequate resolution to determine the location of a fluorescent molecule with high precision. Pixel size: 200 nm. (B) A 2-D Gaussian intensity profile closely approximates the theoretical intensity pattern produced by point-source of light. By selecting a Gaussian intensity profile that best-fits the image, an accurate approximation can be made about the location of the point-source of light (i.e., at the peak intensity of the Gaussian profile).

To statistically fit a 2D Gaussian intensity profile to the intensity profile of a focus, the spot is first isolated from the rest of the image by defining a square region of interest (ROI) that includes the spot and a few pixels beyond, in each direction (Figs. 1B and 3A). Next, an initial estimate of the following parameters must be made: peak intensity, coordinates of the peak, width in each lateral dimension, rotation from horizontal, and background intensity. For peak intensity, the intensity of the brightest pixel is a good initial estimate. For lateral dimensions, the theoretical width of a diffraction-limited spot works well. This is determined as *s*, the standard deviation of a Gaussian distribution, using the following equation.

$$S=0.21\frac{\lambda }{\mathrm{NA}}$$

Here, *λ* is the wavelength of the light that forms the spot, and *NA* is the numerical aperture of the microscope objective.

Lateral symmetry is usually a safe initial assumption, so both dimensions can initially be set equal, but symmetry is never actually true so the two dimensions must be allowed to adjust independently. As with lateral dimensions, it is usually safe to initially assume rotation-from-horizontal as equal to zero and let the algorithm determine the best-fit value. Finally, background intensity is initially estimated as the average intensity of all perimeter pixels – this assumes a clean region of interest, without influence from neighbouring features.

Estimated values are placed in the following equation, and the resulting function evaluated.

$$F(x\text{,}y\text{;}z_{0}\text{,}A\text{,}x_{0}\text{,}y_{0}\text{,}s_{x}\text{,}s_{y})=z_{0}+(A_{e}\left({\left((x=x_{0})/s_{x}\right)}^{2}+{\left((y-y_{0})/s_{y}\right)}^{2}\right)/2$$

Here, *x* and *y* are independent variables and the remaining terms are parameters that are adjusted during fitting: *z*_o_ is the background intensity, *A* is the peak intensity, *x*_o_ and *y*_o_ are the coordinates of the peak, and *s_x_* and *s_y_* are the lateral dimensions (i.e., standard deviations) of the profile in their respective coordinate directions. Initial values for *x*_o_ and *y*_o_ are typically chosen as the coordinates of the center of the pixel with maximum intensity. Regression analysis is run recursively until the solution converges to within a tolerance of 0.01%. Most successful fits converge to a solution with fewer than 10 iterations.

Conceptually, one can imagine plotting the function described by this equation (e.g., Fig. 3B), and comparing its intensity values to those in the image (Fig. 3B). Then, differences are calculated, and the equation’s parameters are iteratively adjusted so as to decrease the difference until the difference, or error, between the function and image is minimized. This process is called ‘minimizing least-squares error’ and is a common statistical method. Once complete, the equation provides a mathematical model of the light emitted by the probes, and the peak of the model function is the best-guess of the location of the source of light – in our case, the FISH probes and site of transcription. The confidence of our estimate (i.e., the precision with which we know the location of the probe), *σ*, is determined using the following equation.

$$\sigma =\left(\frac{s^{2}}{N}+\frac{a^{2}/12}{N}+\frac{8\pi b^{2}s^{2}}{a^{2}N_{2}}\right)$$

Here, *a* is the pixel size in nm, *b* is the amount of background noise in photons/pixel, *N* is the number of photons that contribute to the spot image – estimated as the total signal intensity of the spot before gain is applied, and *s* is the lateral dimension (i.e., one standard deviation).

**Fig. 4:**
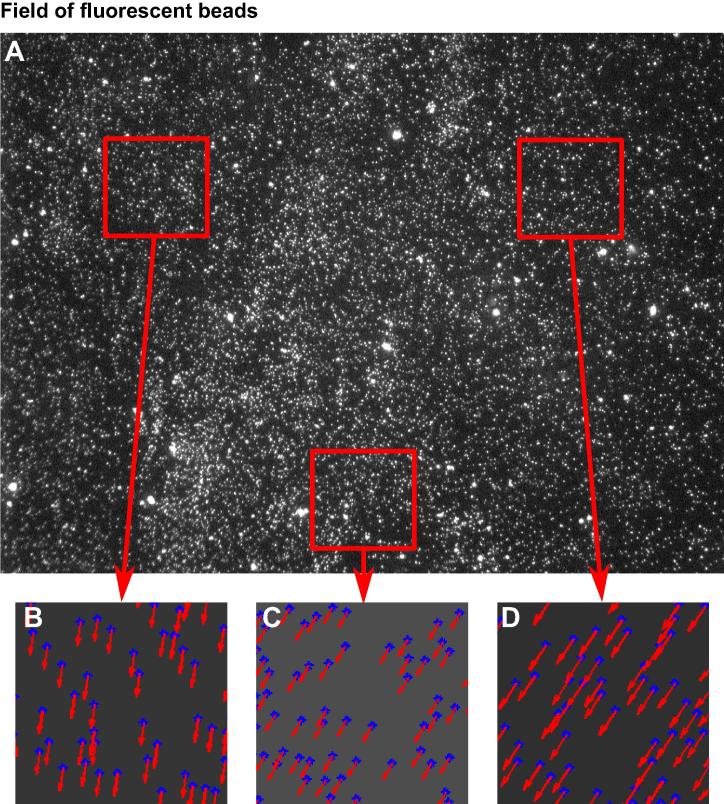
Fluorescent beads as fiduciary markers for inter-channel alignment. The difference in direction and magnitude (B, C, D) of inter-channel misalignment within an image (A) is often dramatic and must be corrected. Microscope objectives attempt to correct for some of this, but residual values can be so great as to dominate the precision of the measurement.

Misalignment between channels is measured by imaging 0.1-μm TetraSpeck beads (Invitrogen). These beads fluoresce in multiple channels, so a single bead can be imaged in each of the channels used for FISH, and the difference between apparent locations of the same bead measured. Beads are adsorbed on to clean coverslips, images acquired, and translational and rotational misalignment measured (e.g., Fig. 4). Misalignment data is used to create a 2D spatial transform (i.e., bi-linear interpolation following a local weighted mean of a minimum of 12 fiduciary points throughout the image), and then applied to mathematically re-align channels. Residual error, post-alignment, is determined by applying the spatial transform to a different image of multi-spectral beads and measuring the remaining difference. This residual error must be included in the total precision of the distance measured between two sets of FISH probes.

To measure the distance between two probes with different colors, the above is performed on each focus viewed in different channels, the geometric distance between positions calculated, and channel misalignment (due to spherical and chromatic aberrations) corrected. The distance measured must be greater than the square root of the sum of the squares of the confidence of the location estimates and the residual channel misalignment.

## 5 An example

In one experiment, we used RNA FISH with pairs of intronic probes – one red and one green – to measure distances between two transcripts copied from different regions of the same gene (*SAMD4A*). Transcripts are copied from these two regions by two different polymerases. A pioneering polymerase initiates ∼10 min after adding TNFα, and then transcribes steadily to reach the terminus after ∼75 min; consequently, this pioneer transcribes regions of the gene lying progressively further down the gene with time (and the terminus is only transcribed after 85 min). As the pioneer transcribes, a second polymerase can initiate at the TSS; however, this soon aborts (for unknown reasons). Repeated initiations and abortions at the TSS ensures that intronic RNAs copied from just this region can be seen from 10 to 85 min after adding the cytokine. By targeting these two regions with FISH probes, we have a method to study the distance between the promoter and the segment being transcribed by the pioneering polymerase.

**Fig. 5:**
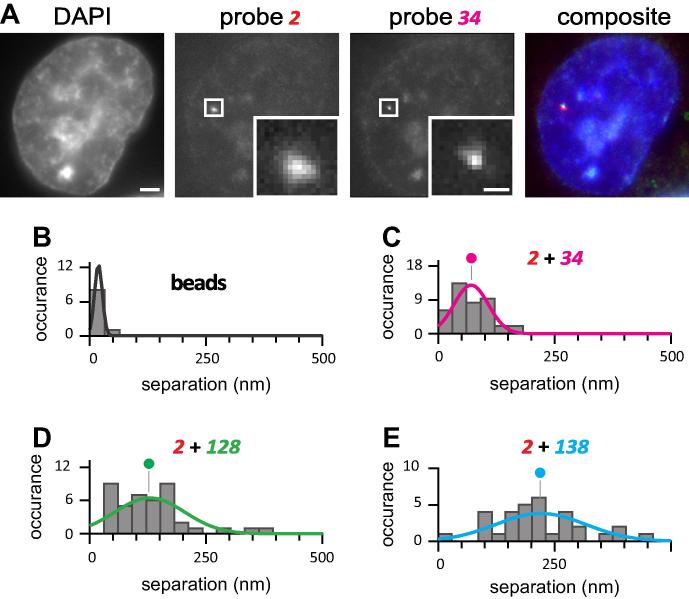
An example, where distance measurements were made between three pairs of FISH probes. (A) Typical images of one nucleus obtained 30 min after stimulation, used for separation measurements. Bar: 2 μm (insets 500 nm, 90-nm pixels). (B) Occurrences given by multispectral 100-nm beads (where the expected separation is zero if channel registration and localization are perfect). (C) The separation (nm) seen between probe 2 (target 2 kbp downstream from the transcription start site) and probe 34 (target 34 kbp downstream). Histograms (30-nm bins) illustrate the number of times a separation was seen (occurrence). Gaussian distributions are fitted to histograms, and normalized by equalizing areas under curves to allow direct comparison of probabilities (circles indicate means). (D) The separation (nm) seen between probe 2 and probe 128 (target 128 kbp downstream). (E) The separation (nm) seen between probe 2 and probe 138 (target 138 kbp downstream).

Results of a typical experiment (using red and green probes) are illustrated in Fig. 5 (adapted from Larkin *et al*., 2013 [29]). A yellow (colocalizing) spot results from targets copied from the same allele (as spot area is so small compared to nuclear area, a green focus can only overlap a red focus copied from a different allele in <1 nucleus in a thousand, assuming random distributions). Note that the use of RNA FISH with intronic probes instead of DNA FISH has the great advantage that it allows us to focus on what can be a minority of active alleles in the population, and not the majority of inactive ones. As the resolution afforded by conventional microscopy is too low to distinguish between nascent transcripts copied from DNA regions lying only 32 kbp apart, we use super-resolution localization to measure (with 30-nm precision) the distance between the red and green foci underlying such yellow foci (like those in Fig. 5A). Two-dimensional Gaussian distributions are fitted to the intensities of the underlying red and green foci, the positions of each peak measured with 15-nm precision, and distances (separations) between peaks measured with 30-nm precision (the increased error results from residual misalignment between channels). Most separations given by red/green fluorescent beads are ⩽30 nm (Fig. 5B), and the distribution seen is that expected of a ‘perfectly’ colocalizing control measured with 30-nm precision. Probe pairs targeting transcripts copied from the regions that are 2 and 34 kbp from the TSS yield a range of separations, with a mean of 68 nm (Fig. 5C). Probes targeting transcripts from the 2 and 128 kbp regions give a pattern with a slightly greater separation (Fig. 5D). However, pairs targeting transcripts from the 2 and 138 kbp regions give much greater separations (Fig. 5D). Clearly, the degree of separation does not increase uniformly with time, as would be expected if the pioneer was tracking steadily down the long gene away from the succession of polymerases transcribing the TSS. After repeating this experiment with more probes pairs at different locations along the gene, we confirmed this behaviour. These results are not consistent with any model for transcription that involves the polymerases tracking along their templates when active (whether they initiated in a “factory” or not). Rather, they are consistent with the two active polymerases being immobilized in the same (early on) or different (later) transcription “factories”, and with those fixed polymerases generating their transcripts as they reel in their templates. As the arguments are not of direct interest here, and as they have been reviewed in Larkin *et al*., 2013 [29], they will not be discussed further here.

## 6 Concluding remarks

The method described here provides a means of determining the relative geometric distance between sites of gene transcription using RNA FISH. It involves, first, labeling nascent transcripts with probes targeting intronic regions (which mark transcription sites). Next, these probes are hybridized to (nascent) RNA. Finally, images are acquired – typically on a relatively low-tech widefield fluorescence microscope – and processed so as to allow measurements of distance as short as 20 nm.

Various parts of this approach are challenging. First, a good biological system should be selected. Some genes can be highly active so that most alleles in the population are being transcribed at any one moment (e.g., globin genes in erythroid precursor cells). However, many other so-called “active” genes are not nearly so active. Even with our system (under optimal conditions), roughly one-third of the cells in the population possess no active *SAMD4A* allele (detectable by RNA FISH), and the inactive cells inevitably contribute to the background. Moreover, our system provides an excellent gene switch, so that “active” and “inactive” cells only a few minutes distant in time can be compared – and this provides further confidence that the foci seen are real ones (and not due to background). Second, FISH probes must be designed carefully. They must hybridize specifically, and be short enough to penetrate the nucleus whilst being long enough to provide enough signal. Probes used in the experiments described here emit long-wavelength fluorescence, but ones fluorescing at shorter wavelengths could be used equally well, as long as probe sets remain spectrally distinct. Third, many biologists find the image processing required for super-resolution measurements demanding. This challenge is compounded if measurements are made in 3D (and not 2D) nuclear space (e.g., using a confocal microscope). Nevertheless, we have successfully used the method to study the spatial conformation of one long gene [29], and the distance of that long gene from others that respond similarly [9], [30]. As compared to techniques that are colloquially referred to as super-resolution microscopy (e.g., STORM, [31]), the methods described herein to localize probes with super-resolution accuracy are the same. However, the images obtained are more intuitively connected to the ones we see with our own eyes through a fluorescence microscope; moreover, successive photo-activation and bleaching is not required to spatially separate fluors of the same wavelength, as more than one probe per nucleus is rarely observed.
